# MVA-HBVac—A novel vaccine vector that allows pan-genotypic targeting of hepatitis B virus by therapeutic vaccination

**DOI:** 10.1016/j.omtn.2025.102641

**Published:** 2025-07-23

**Authors:** Anna D. Kosinska, Martin Kächele, Helene A. Kerth, Martin Mück-Häusl, Edanur Ates Öz, Merve Gültan, Lea Hansen-Palmus, Julia Sacherl, Chunkyu Ko, Julia Festag, Michael H. Lehmann, Carolin Mogler, Katja Steiger, Percy A. Knolle, Tanja Bauer, Asisa K. Volz, Ulrike Protzer

**Affiliations:** 1 Institute of Virology, School of Medicine and Health, Technical University of Munich/Helmholtz Munich, 81675 Munich, Germany; 2 Division of Virology, Institute for Infection Medicine and Zoonoses, Department of Veterinary Sciences, Ludwig-Maximilians-University Munich (LMU Munich), 80539 Munich, Germany; 3 Institute of Pathology, School of Medicine and Health, Technical University of Munich, 81675 Munich, Germany; 4 Institute of Molecular Immunology, School of Medicine and Health, Technical University of Munich, TUM University Hospital Rechts der Isar, 81675 Munich, Germany; 5 German Center for Infection Research (DZIF), Partner Site Munich, 81675 Munich, Germany

**Keywords:** MT: Delivery Strategies, HBV, chronic hepatitis B, therapeutic vaccination, *TherVacB*, MVA, viral vector, heterologous prime boost

## Abstract

Therapeutic vaccination holds the promise to cure chronic hepatitis B virus (HBV) infection. We hypothesize that B cell, CD4, and CD8 T cell responses are necessary to overcome HBV-specific immune tolerance in chronic infection because they accompany the rare, spontaneous resolution of chronic HBV infection. Therefore, we designed the heterologous prime-boost vaccine *TherVacB* in which virus-like particle vaccination stimulates B and helper CD4 T cells and primes cytotoxic effector CD8 T cells and a vector boost expands the T cell response. Here, we report the generation and characterization of a novel modified vaccinia virus Ankara (MVA)-based vector, MVA-HBVac, capable of inducing strong and multi-specific T cell responses against the immunodominant epitopes of four different viral proteins covering >95% of HBV strains circulating worldwide. When MVA-HBVac was administered after a prime with adjuvanted hepatitis B S- and core-antigens forming virus-like particles, it activated strong HBV-specific CD4 and CD8 T cell responses against the major HBV antigens *in vivo* in naive and HBV carrier mice. This induced a sustained antiviral effect against different, clinically relevant HBV genotypes. Our data showed that the *TherVacB* regimen employing the novel, pan-genotypic MVA-HBVac vector could overcome HBV-specific immune tolerance and lead to the initiation of clinical trials evaluating the therapeutic vaccine.

## Graphical abstract

**Figure:**
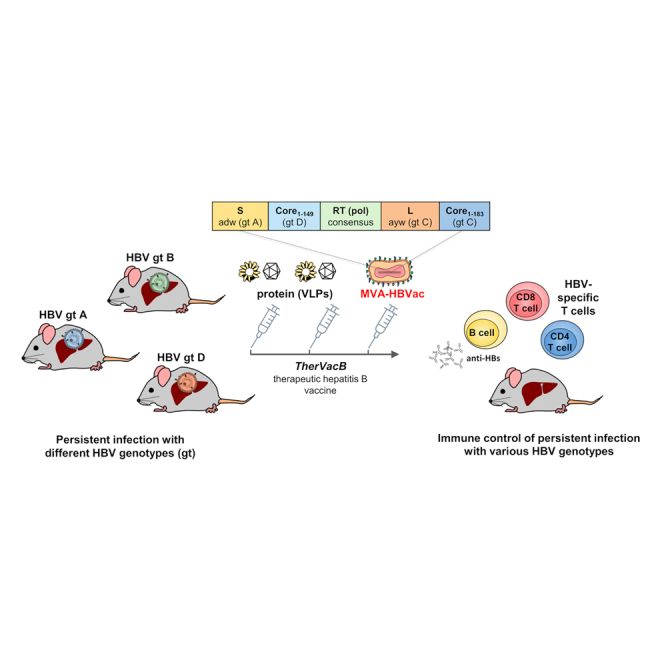

## Teaser

Ulrike Protzer and colleagues developed a novel therapeutic hepatitis B vaccine vector, MVA-HBVac, designed to target 95% of circulating HBV isolates worldwide. Administration of MVA-HBVac following two protein prime immunizations in the *TherVacB* vaccination scheme induced strong HBV-specific T cell responses and reduced or even “cured” HBV in mice carrying various HBV genotypes.

## Introduction

Hepatitis B virus (HBV) chronically infects 250 million humans and causes more than 1 million deaths per year due to HBV-associated liver cirrhosis and hepatocellular carcinoma.^1^ Current treatment of chronic hepatitis B (CHB) mostly relies on nucleos(t)ide analogs, which efficiently suppress viral replication but do not eliminate the virus from infected hepatocytes and only slightly reduce the risk for the development of liver cancer. Many patients, therefore, require long-term therapy, causing high costs and compliance problems. The treatment with pegylated interferon (IFN) alpha may result in viral clearance in a low percentage of patients treated, but the treatment is limited by systemic side effects.^2^ Therefore, novel, curative treatment strategies for CHB are urgently needed. There is convincing evidence that HBV persistence in infected individuals is strongly associated with developing a virus-specific immune tolerance characterized by scarce and dysfunctional HBV-specific T cells.^2^ Developing strong and polyclonal T cell responses, in contrast, leads to HBV clearance.^3^ Patients suffering from CHB who received an allogenic bone marrow transplantation from individuals with resolved HBV infection could clear the virus, indicating the major impact of cellular immunity on the cure of hepatitis B.^4^ Furthermore, individuals who resolve CHB develop a strong and specific T cell response that persists after the elimination of the virus.^5^ Recent studies indicated an important role of CD4 T cell responses during spontaneous resolution of CHB.^6^ Based on these results and the identified connection between the host’s T cell response and the course of an HBV infection, stimulation of host immunity by therapeutic vaccination is regarded as a promising treatment option. However, attempts at re-stimulating the immune systems of CHB patients to evoke anti-HBV immunity using protein-based therapeutic vaccines have not led to the desired outcome so far, indicating the need to develop vaccines capable of inducing more potent immune responses.^7^

Employing heterologous prime-boost strategies is one of the most promising approaches to increasing the immunogenicity of protein-based vaccines against difficult targets and diseases like chronic infection or cancer that require overcoming immune tolerance.^8^ Combining a priming step with a boost using recombinant modified vaccinia Ankara (MVA) viral vectors showed the induction of superior T cell immunity when compared to a single prime vaccination in chimpanzees.^9^ MVA is a highly attenuated poxvirus vector used as a prototype vaccine to eradicate human smallpox. The unique characteristics of MVA, such as safety, efficient gene expression, and the ability to induce strong antigen-specific immune responses, make the vector an excellent candidate for vaccine development.^10^ Its efficacy has been demonstrated in humans.^11^^,^^12^^,^^13^ We have previously developed a protein-prime, MVA-boost vaccination, termed *TherVacB*, which was able to induce HBV-specific antibody and T cell responses in preclinical animal models of persistent HBV infection, i.e., HBV carrier mice that are either HBV-transgenic or have been transduced with an adeno-associated virus transferring a replication-competent HBV genome (AAV-HBV), in which strong HBV-specific immune tolerance exists from persistent exposure of the host’s immune system to viral antigens.^14^^,^^15^^,^^16^^,^^17^ Using protein antigens for prime vaccination that form virus-like particles (VLP) and are combined with an adjuvant that permits a T helper type 1 (Th1)-type CD4 T cell response enabled successful therapeutic vaccination.^17^ These encouraging results served as the starting point for translating *TherVacB* into the clinics.

Developing a therapeutic vaccine that can induce broad HBV-specific immune responses targeting the most commonly circulating genotypes and serotypes is crucial for a worldwide application, but it has been poorly addressed. Therefore, in this study, we aimed to design an optimal vaccine vector termed MVA-HBVac, which would be suitable for clinical translation and cover the main HBV serotypes and genotypes circulating worldwide. The vector was characterized *in vitro* to ensure purity, functionality, and stability and tested for its efficacy *in vivo* in naive C57BL/6 mice and in an AAV-HBV mouse model carrying HBV strains of genotype (gt) A, B, C, or D, covering the antigens of the most prevalent genotypes worldwide.^18^

## Results

### Generation and characterization of the novel vaccine vector MVA-HBVac for *TherVacB* boosting

**Figure 1:**
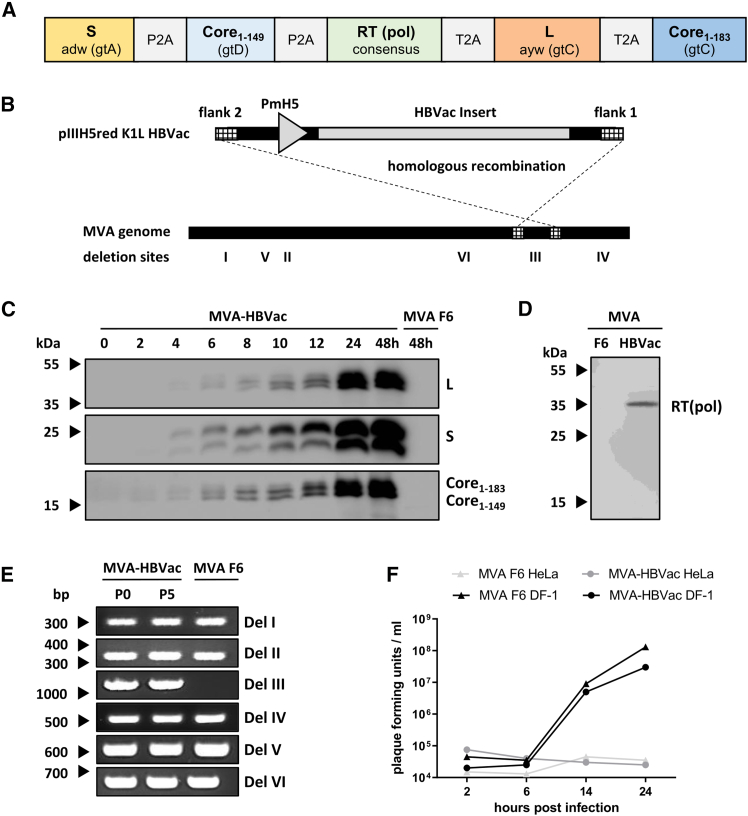
Generation and characterization of MVA-HBVac (A) The MVA-HBVac expression cassette encodes five HBV proteins (S, subtype adw, gtA; truncated Core_1-149_, gtD; RT domain of the viral polymerase [RT(pol)]; consensus sequence of L, subtype ayw, gtC; full-length Core_1-183_, gtC) linked by P2A and T2A sites. (B) MVA-HBVac was created via homologous recombination between the MVA F6 genome and the transfer plasmid pIIIH5redMVA-HBVac, as depicted in the scheme. (C and D) Expression of (C) glycosylated and non-glycosylated S proteins (24 and 27 kDa) and L proteins (39 and 42 kDa, respectively); full-length (C_1–183_) and truncated (C_1–149_) versions of core protein, and (D) RT(pol) was detected by western blotting of DF-1 cell lysates after MVA-HBVac infection at a multiplicity of infection (MOI) of 1 plaque-forming unit (pfu) per cell. DF-1 cells infected by MVA F6 (MOI 1 pfu/cell) were controls. (E) Genome stability of MVA-HBVac over five low-titer passages (MOI 0.01 pfu/cell) in DF-1 cells was analyzed by PCR. The MVA F6 genome was used as a control. (F) The viral growth of MVA-HBVac was analyzed in DF-1 and HeLa cells. The MVA F6 wild-type strain was used as a control (MOI 1 pfu/cell). Viral titers were determined at the indicated time points by a plaque assay.

To improve the strength and breadth of HBV-specific immune responses elicited by *TherVacB,* we designed a polycistronic MVA vector, MVA-HBVac, which encodes five HBV proteins: the small envelope proteins S (subtype adw, gtA), the large envelope protein L (ayw, gtC), the full-length core protein forming the HBV capsid Core_1-183_ (gtC), a C-terminally truncated version of the Core protein Core_1-149_ (gtD), and a consensus sequence of the reverse transcriptase (RT) domain of the viral polymerase (pol) (Figure 1A). The sequences included in the HBVac insert were selected based on the T cell responses observed during self-limiting hepatitis B, which usually target the viral surface and capsid proteins as well as the viral polymerase. The included sequences were optimized to cover the epitopes of the most prevalent HBV genotypes but kept identical to naturally occurring viral sequences to ensure correct folding and processing. This is necessary for efficient antigen presentation on infected cells and the secretion of highly immunogenic, non-infectious subviral particles.^19^ The coding sequences in the HBVac insert are linked by P2A and T2A sites selected according to their reported *in vivo* efficacy to allow the expression of all five proteins encoded by the polycistronic insert.^20^ P2A and T2A sites were partially codon-optimized to avoid recombination.

A previously described protocol^21^ was used and applied under GMP-conform conditions to create a recombinant MVA vector suitable for clinical testing. The HBVac fragment was cloned into the shuttle vector pIIIH5redK1L, which was used to insert it into the deletion site III of the MVA F6 genome by homologous recombination (Figure 1B). Expression of the HBV proteins was confirmed by infecting DF-1 cells with MVA-HBVac and analyzing protein content over 48 h by western blotting (Figure 1C). Infected cells showed expression of the expected proteins S, L, Core_1-149_, and C_1-183_, indicating the full functionality of the recombinant MVA. The RT(pol) protein was detected by western blotting after 24 h (Figure 1D). S and L envelop proteins showed the expected glycosylation patterns, indicating correct protein processing essential for the secretion of subviral particles and antigen presentation.^22^ Correct integration of the HBVac fragment into the genome of MVA and purity of MVA-HBVac was confirmed by PCR. Additionally, we demonstrate that MVA-HBVac is stable even after five low-titer passages (Figure 1E). Analyzing the growth kinetics of MVA-HBVac compared to the parental MVA F6 ensured efficient replication in DF-1 cells and replication deficiency in mammalian HeLa cells, allowing for safe virus handling under S1 conditions (Figure 1F). Taken together, these data indicate that the polycistronic MVA-HBVac vaccine vector was successfully generated.

### The *TherVacB* regimen, including an MVA-HBVac boost, elicits vigorous and multi-specific immune responses in HBV-naïve mice

**Figure 2:**
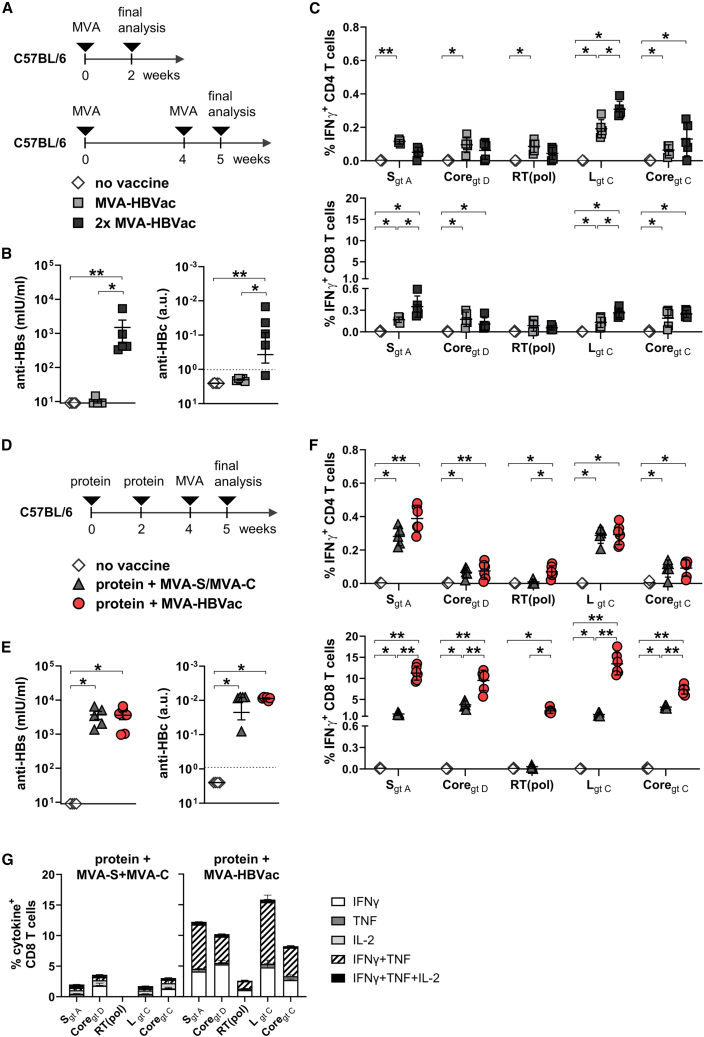
Immunogenicity of MVA-HBVac in HBV-naïve mice (A) C57BL/6 mice (*n* = 5) received one or two vaccinations with MVA-HBVac at week 0, or 0 and 4. Vaccine-elicited HBV-specific antibody and T cell responses were analyzed 2 weeks after a single vaccination or 1 week after the boosting (week 5). Mice receiving no vaccinations served as controls. (B) Anti-HBs and anti-HBc levels detected in mouse serum at week 5. (C) Frequencies of HBV-specific IFNγ^+^ CD4 (upper panel) and IFNγ^+^ CD8 T cells (lower panel) detected by intracellular cytokine staining (ICS) of isolated splenocytes after 16 h *ex vivo* stimulation with five overlapping peptide pools covering the proteins expressed by MVA-HBVac. (D) C57BL/6 mice (*n* ≥ 4) received a prime vaccination with 20 μg CpG-adjuvanted, particulate HBsAg and HBcoreAg at weeks 0 and 2. At week 4, animals received a vector-boost vaccination with either MVA-HBVac or a mixture of MVA-S and MVA-C. Mice receiving no vaccinations served as controls. One week after the boost, vaccine-elicited HBV-specific antibody and T cell responses were analyzed. (E) Anti-HBs and anti-HBc levels detected in mouse serum at week 5. (F). Frequencies of HBV-specific IFNγ^+^ CD4 (upper panel) and IFNγ^+^ CD8 T cells (lower panel) detected by intracellular cytokine staining (ICS) of isolated splenocytes after 16 h *ex vivo* stimulation with five overlapping peptide pools covering the proteins expressed by MVA-HBVac. (G) Fraction of CD8 T cells staining positive for indicated cytokines after *ex vivo* stimulation with HBV-specific overlapping peptide pools. Mean ± standard error of mean is shown. Statistical analyses were performed using Kruskal-Wallis test with Dunn’s multiple comparison correction. Asterisks mark statistically significant differences: ∗*p* < 0.05; ∗∗*p* < 0.01.

To test its immunogenicity *in vivo*, naive C57BL/6 mice were immunized with MVA-HBVac once at week 0 or twice at weeks 0 and 4 (Figure 2A). Mice receiving no vaccine served as controls. Vaccine-induced HBV-specific antibody and T cell responses were determined at week 2 after one MVA-HBVac vaccination or at week 5 after two MVA-HBVac vaccinations.

Single vaccination of mice with MVA-HBVac did not induce any significant anti-HBs or anti-HBc antibodies (Figure 2B). Applying MVA-HBVac twice in a homologous prime-boost regimen significantly improved antibody responses and elicited moderate levels of anti-HBs and anti-HBc. To determine the breadth of T cell responses, we used overlapping peptide pools covering the HBV antigens from different HBV genotypes encoded by the HBVac insert. One vaccination with MVA-HBVac already elicited detectable HBV-specific CD4 T cell responses directed against all MVA-HBVac encoded antigens compared to non-vaccinated animals. Still, additional vaccination with MVA-HBVac did not increase the frequencies of IFNγ^+^ CD4 T cells (Figure 2C, upper panel). Although both regimens elicited IFNγ^+^ CD8 T cell responses against envelope and core proteins, their magnitudes remained relatively low (Figure 2C, lower panel). None of the regimens elicited a significant RT(pol)-specific CD8 T cell response in the spleens of the vaccinated mice.

To improve the induction of HBV-specific CD8 T cell responses, a prerequisite for successful therapeutic vaccination, we primed immune responses according to the *TherVacB* heterologous protein-prime, MVA-boost vaccination scheme using adjuvanted HBsAg and HBcoreAg. MVA-HBVac was used as the booster component. The performance of the new vector was compared to the previously used mixture of MVA-C and MVA-S vectors expressing either HBV core or S proteins. Groups of mice received the prime vaccination with HBcoreAg and HBsAg twice, at weeks 0 and 2, and two weeks later, at week 4, were boosted either with a mixture of MVA-S and MVA-C or the newly developed MVA-HBVac (Figure 2D). Mice receiving no vaccine served as controls. Vaccine-induced HBV-specific antibody and T cell responses were determined 1 week after the boost vaccination.

After priming with adjuvanted proteins, boost vaccination with MVA-HBVac led to anti-HBs and anti-HBc responses comparable to those detected in mice boosted with the MVA-S and MVA-C combination (Figure 2E), but 1log_10_ higher anti-HBs titers and 2log_10_ higher anti-HBc titers than the homologous MVA-HBVac regimen (Figure 2B). In addition, boosting vaccination with MVA-HBVac or the MVA-S and MVA-C combination elicited comparable IFNγ^+^ CD4 T cell responses directed against all encoded antigens (Figure 2F, upper panel; Figures S1A and S1B). CD4 T cell responses against core proteins and RT(pol) were comparable after homologous and heterologous vaccination but more pronounced against S after the heterologous protein-prime, MVA-boost. Most importantly, priming with adjuvanted proteins considerably improved HBV-specific CD8 T cell responses detected after MVA-boost vaccination (Figure 2F, lower panel; Figure S1C). Boosting with MVA-HBVac elicited significantly more S-, Core_gt D_-, L-, and Core_gt C_-specific IFNγ^+^ CD8 T cells than combining MVA-S and MVA-C. As expected, vaccination with MVA-HBVac induced an RT(pol)-specific CD4 and CD8 T cell response, but the combination of MVA-S and MVA-C did not. Interestingly, RT(pol)-specific CD8 T cell responses were more pronounced after protein- than after MVA-HBVac priming, although no RT(pol) protein was provided (Figures 2C and 2F). In comparison to a boost with MVA-S and MVA-C, boosting with MVA-HBVac significantly increased the frequencies of polyfunctional IFNγ and tumor necrosis factor (TNF) double-positive HBV-specific CD8 T cells detected after stimulation with all five virus-derived peptide pools (Figure 2G), confirming the excellent immunogenicity of MVA-HBVac *in vivo*.

Together, these results demonstrate that employing MVA-HBVac as a part of a heterologous protein-prime, MVA-boost vaccination scheme considerably improves HBV-specific immunity. *TherVacB* vaccination with MVA-HBVac elicited comparable antibody and HBV-specific CD4 T cell responses against S and core as separate MVA vectors expressing only a single antigen. However, it induced stronger, broader, and more functional CD8 T cell responses.

### The *TherVacB* regimen employing MVA-HBVac for boost vaccination induces long-term immunity in HBV carrier mice

To investigate whether the *TherVacB* regimen employing the novel MVA-HBVac vector can induce satisfactory HBV-specific immune responses during persistent viral replication, we performed *TherVacB* vaccination using MVA-HBVac in an AAV-HBV mouse model. In the AAV-HBV mouse model, recombinant AAV carrying a replication-competent HBV genome infects mouse hepatocytes where the AAV-HBV persists and serves as a template for the transcription of HBV RNAs translated into HBV proteins and initiating HBV replication. This eventually establishes an HBV-specific immune tolerance, mimicking the human chronic HBV carrier state.^15^^,^^23^^,^^24^ Although mice do not support the intrahepatic spread of HBV, the AAV-HBV model offers the possibility to examine therapeutic vaccination strategies in immunocompetent mice in a setting resembling human chronic infection.

**Figure 3:**
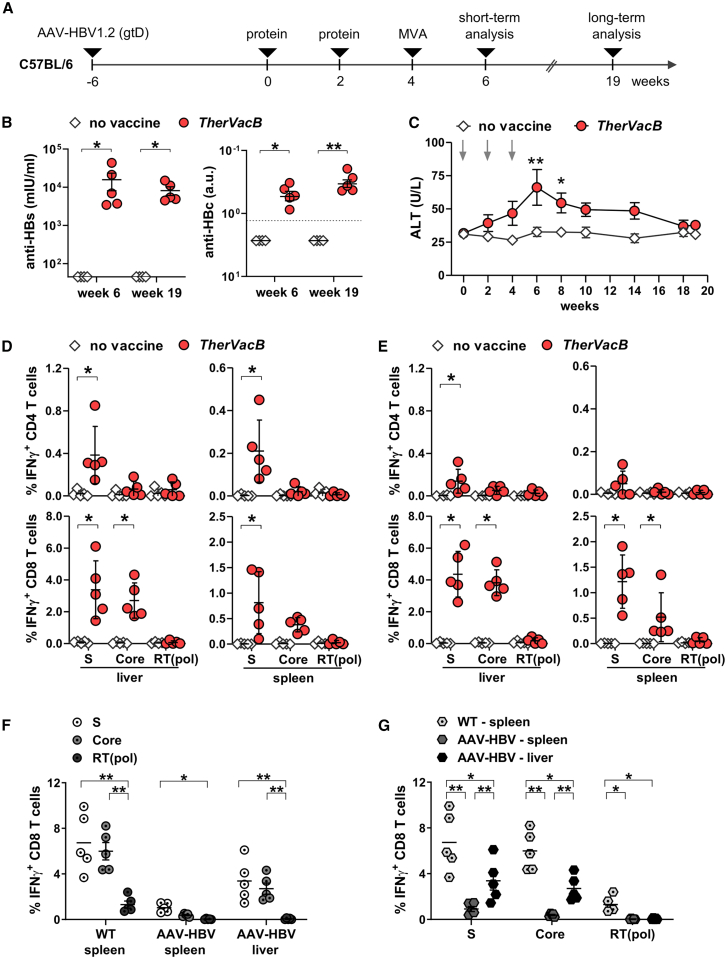
Immunogenicity of *TherVacB* using an MVA-HBVac boost in HBV-carrier mice (A) C57BL/6 mice (*n* = 5) were intravenously injected with 4 × 10e9 GE of an AAV vector carrying a 1.2-fold overlength HBV genome of gtD (AAV-HBV). *TherVacB* vaccination was initiated 6 weeks after AAV-HBV infection (week 0). Prime vaccination with CpG-adjuvanted, HBsAg, and HBcoreAg VLPs was performed at weeks 0 and 2 and boost vaccination with MVA-HBVac at week 4. Mice receiving no vaccinations served as controls. Mice were sacrificed 2 or 15 weeks after the MVA-HBVac boost (weeks 6 and 19, respectively). (B) Anti-HBs and anti-HBc titers were measured in mouse sera. (C) Time kinetics of serum ALT levels. (D and E) HBV-specific T cell responses were determined in lymphocytes isolated from spleen and liver by intracellular cytokine staining (ICS) after 16 h *ex vivo* stimulation with three optimized overlapping peptide pools. (D) Frequencies of S-, core-, and RT(pol)-specific IFNγ^+^ CD4 and CD8 T cells detected at week 6. (E) Frequencies of S-, core-, and RT(pol)-specific IFNγ^+^ CD4 and CD8 T cells detected at week 19. (F and G) Naive C57BL/6- and AAV-HBV-infected mice were immunized according to the heterologous protein prime, MVA-HBVac boost *TherVacB* scheme. One week after the MVA-HBVac (week 5), mice were sacrificed, and HBV-specific CD8 T cell responses were determined in the spleen and liver. As WT mice express no target antigen in the liver, only data from the spleen are depicted. (F) Comparison of the magnitude of S-, core-, and RT(pol)-specific IFNγ^+^ CD8 T cell response per vaccination group. (G) Comparison of the magnitude of S-, core-, and RT(pol)-specific IFNγ^+^ CD8 T cell responses in each group. Mean ± standard error of mean is shown. Statistical analyses were performed using (B, D, and E) Mann-Whitney test and (C, F, and G) two-way ANOVA. Asterisks mark statistically significant differences: ∗*p* < 0.05; ∗∗*p* < 0.01.

C57BL/6J mice were infected with AAV-HBV-1.2 (gtD) 6 weeks before vaccination to establish persistent HBV replication and HBV-specific immune tolerance. AAV-HBV mice received two protein vaccinations with adjuvanted VLPs, HBsAg, and HBcoreAg at weeks 0 and 2 and a booster with MVA-HBVac at week 4 (Figure 3A). AAV-HBV mice receiving no vaccine served as controls. The animals were sacrificed either 2 weeks after the boost (week 6) to analyze vaccine-induced immune responses or 15 weeks after the boost (week 19) to study the long-term antiviral effect of the vaccination.

Two weeks after the MVA-HBVac boost, all immunized mice had developed high anti-HBs and anti-HBc titers in their serum and maintained a strong anti-HBs response until the final analysis at week 19 (Figure 3B). Monitoring of ALT activity in mouse serum revealed mild flares shortly after MVA-HBVac vaccination, at weeks 6 and 8, indicating the peak of effector T cell response and lysis of hepatocytes by HBV-specific CD8 T cells (Figure 3C). Importantly, ALT levels normalized until week 10, indicating only transient MVA-HBVac-mediated liver damage. No weight loss was observed (Figure S2).

At weeks 6 and 19, we isolated lymphocytes from the spleen and liver and analyzed HBV-specific CD4 and CD8 T cell responses by intracellular cytokine staining (ICS). Since the sequences of the different HBV genotypes largely overlap and no significant differences in stimulation with S and L or Core_gtD_ and Core_gtC_ peptide pools were observed in HBV-naïve mice, we decided to use optimized peptide pools comprising the three major HBV immunogens—S of gtA/D, Core of gtA/C/D (full-length), and RT(pol) (consensus sequence) for *ex vivo* stimulation of T cells.

At week 6, we detected significant S-specific IFNγ^+^ CD4 T cell responses in the livers and spleens of all mice receiving *TherVacB* (Figure 3D, upper panel). Frequencies of core- or RT(pol)-specific IFNγ^+^ CD4 T cells were only marginally increased. Analysis of the HBV-specific CD8 T cell response in the spleen and liver revealed significant frequencies of IFNγ^+^ CD8 T cells directed against S and Core (Figure 3D, lower panel). The majority of HBV-specific IFNγ^+^ CD8 T cells were localized in the liver rather than in the spleen, indicating efficient T cell migration to the liver, where HBV replicates and HBV antigens are expressed. In contrast to the results observed in HBV-naïve mice, no RT(pol)-specific CD8 T cells could be detected in the AAV-HBV mice. Although the frequencies of IFNγ^+^ S-specific CD4 T cells dropped from week 6 to week 19, vigorous S- and core-specific effector CD8 T cell responses could be detected in the livers and spleens of mice receiving *TherVacB* even 15 weeks after the MVA-HBVac boost (Figure 3E).

To determine how *TherVacB*-induced CD8 T cell responses compare between HBV-naïve and carrier mice, we immunized wild-type C57BL/6 (WT) and HBV-carrier mice and analyzed CD8 T cell responses 1 week after MVA-boost. As WT mice have no target antigen in their livers, no liver-associated, HBV-specific T cells were detected (data not shown). *TherVacB* induced significant S- and core-specific CD8 T cell responses in WT mice, but a much lower RT(pol)-specific response (Figure 3F), because the prime vaccination only contains S and core but no polymerase antigens. Overall, the T cell response in the spleen was reduced in HBV-carrier mice compared to WT mice but was targeted to the liver. In the livers of HBV-carrier mice, we observed similar frequencies of S- and core-specific but no RT(pol)-specific CD8 effector T cells (Figures 3F and 3G). In the spleens, representing the reservoir of circulating T cells, only S-specific CD8 T cells were detected in HBV carriers at low frequency (Figure 3F). This analysis indicates that *TherVacB* induces a similar dominance pattern of HBV-specific CD8 T cell response in naive and HBV-carrier mice. T cell responses appear to be reduced in HBV carriers, but a comparison is difficult due to the divergent organ distribution.

Taken together, boosting with MVA-HBVac following protein priming elicited a sustained antibody response and S- and core-specific effector T cell responses that are targeted to the liver in HBV carrier mice.

### The MVA-HBVac-containing *TherVacB* regimen controls persistent HBV replication

**Figure 4:**
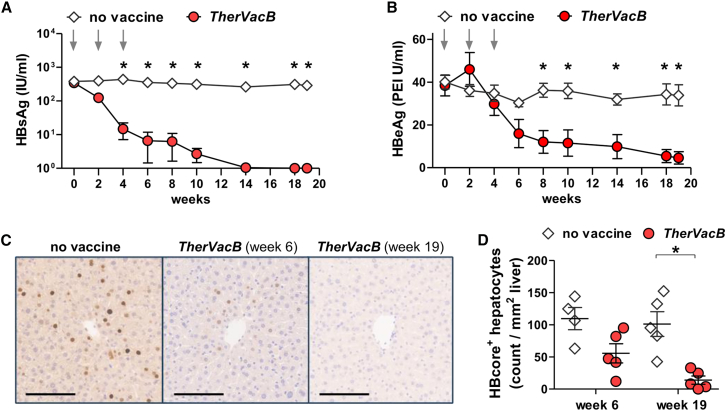
Antiviral effect of *TherVac*B using an MVA-HBVac boost in HBV-carrier mice C57BL/6 mice (*n* = 5) were intravenously injected with 4 × 10e9 GE of an AAV vector carrying a 1.2-fold overlength HBV genome of gtD (AAV-HBV). *TherVacB* vaccination was initiated 6 weeks after AAV-HBV infection (week 0), and mice were sacrificed 15 weeks after the MVA-HBVac boost (week 19). (A and B) Time kinetics of serum (A) HBsAg and (B) HBeAg levels. (C) Representative images of liver immunohistochemistry staining for core protein (brown). Scale bars, 100 μm (D) Quantification of the numbers of core-positive hepatocytes per mm^2^. Mean ± standard error of mean is shown. Statistical analyses were performed using (D) Mann-Whitney test and (A and B) two-way ANOVA with Bonferroni multiple comparison correction. Asterisks mark statistically significant differences: ∗*p* < 0.05.

To investigate whether the vigorous HBV-specific immunity induced by *TherVacB* can result in a sustained antiviral effect in HBV-carrier mice, the impact of vaccination on viral parameters was assessed. Serum HBsAg and HBeAg levels were monitored until the final analysis at week 19. Serum HBsAg levels started to decrease after the vaccination with adjuvanted HBsAg, while they remained unchanged in unvaccinated mice (Figure 4A). This effect could be attributed to the robust induction of anti-HBs antibodies by the protein priming of *TherVacB* (Figure 3B). A decline in HBeAg levels, however, was only observed after the boost vaccination with MVA-HBVac (Figure 4B). The decrease in HBeAg levels was accompanied by elevated ALT activity, indicating that, unlike the antibody-mediated HBsAg reduction, the loss of HBeAg is mainly caused by the cytotoxic CD8 T cells in the liver expanded by MVA-HBVac. During the observation period, secreted HBV antigens in the blood of vaccinated animals dropped continuously, resulting in a drop of HBsAg by more than 2log10 and an approximately 5-fold reduction of HBeAg until week 19. Accordingly, a continuous decrease in core-positive hepatocytes was observed in the livers of the mice receiving *TherVacB*, resulting in a 90% reduction at week 19 compared to unvaccinated controls (Figures 4C and 4D).

Taken together, including MVA-HBVac as a booster vaccination into the *TherVacB* scheme, elicited sustained HBV-specific immunity and resulted in the immune control of persistent HBV replication and efficient elimination of HBV-positive hepatocytes. In addition, vaccination with MVA-HBVac was well tolerated, and no extensive liver damage was observed in any of the *TherVacB*-immunized mice.

### The establishment of mouse models to study therapeutic vaccination against different HBV genotypes

Having demonstrated the strong therapeutic effects upon boosting with the novel MVA-HBVac vector against persistent infection with HBV of gtD, we wondered whether MVA-HBVac could break immune tolerance specific to other prevalent HBV genotypes. To investigate this, we generated recombinant AAVs encoding 1.3-overlength HBV genomes of gtA, gtB, gtC, and gtD.^25^ We first determined whether one can establish long-term HBV replication of the different HBV genotypes in immunocompetent mice. We transduced immunocompetent C57BL/6 mice with AAV-HBV-gtA, -gtB, -gtC, and -gtD, aiming at comparable HBsAg levels, and then followed the markers of HBV infection in the serum for 17 weeks.

**Figure 5:**
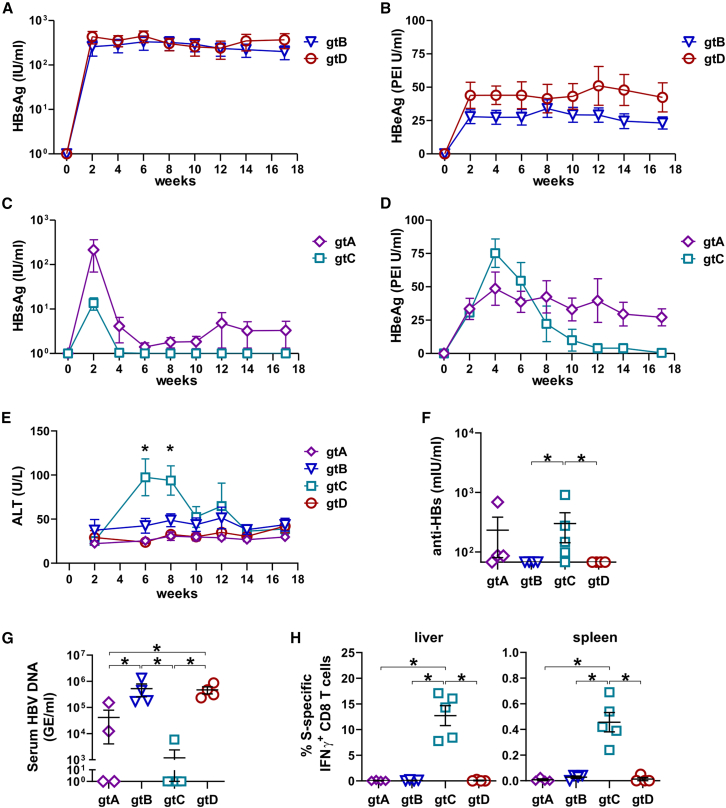
Characterization of the course of AAV-HBV infection with the most prevalent HBV genotypes Six- to eight-week-old C57BL/6 mice (*n* ≥ 4) were intravenously injected with AAV vectors carrying a 1.3-fold overlength HBV genome of gtA (1.5 × 10e10 GE), gtB (1.9 × 10e9 GE), g C (5. 5 × 10e9 GE), or gtD (4 × 10e9 GE) and monitored for 17 weeks. (A and B) Time kinetics of (A) HBsAg and (B) HBeAg in serum after infection with AAV-HBV-gtB and -gtD. (C and D) Time kinetics of serum (C) HBsAg and (D) HBeAg after infection with AAV-HBV-gtA and -gtC. (E) Time kinetics of serum alanine transaminase (ALT) in all infected mice. (F) Anti-HBs titers were measured in mouse serum at week 17. (G) Quantification of HBV-DNA in serum at final analysis. (H) Frequencies of S-specific IFNγ+ CD8 T cells detected by ICS in liver-associated and splenic lymphocytes after 16 h *ex vivo* stimulation with the S-specific overlapping peptide pool at week 17. Mean ± standard error of mean is shown. Statistical analyses were performed using (E) two-way ANOVA with Bonferroni multiple comparison correction and (F–H) Kruskal-Wallis test with Dunn’s multiple comparison correction. Asterisks mark statistically significant differences: ∗*p* < 0.05. GE-genome equivalents.

Hereby, we found that HBV replication kinetics varied significantly between the different genotypes (Figures 5A–5D). Only mice infected with AAV-HBV-gtB and -gtD had constant serum HBsAg and HBeAg levels throughout the monitoring period (Figures 5A and 5B). Still, HBV gtB carrier mice had lower serum HBeAg levels than HBV gtD carrier mice (Figure 5B).

HBsAg levels in mice infected with AAV-HBV-gtA and -gtC peaked at week 2 post-infection and then decreased to a stable, low level for gtA but became undetectable for gtC (Figure 5C). After AAV-HBV-gtA infection, serum HBeAg levels reached a plateau around week 2 post-infection and thereafter remained relatively stable, indicating that with HBV gtA, a carrier state can be achieved (Figure 5D). In mice replicating HBV gtC, HBeAg values increased 3-fold between weeks 2 and 4 and then rapidly dropped and reached undetectable levels in all mice by week 17 post-infection (Figure 5D).

A significant ALT increase at weeks 6 and 8 post-infection accompanied the HBeAg decrease in mice replicating HBV gt C. In contrast, mice transduced with HBV gtA, gtB, and gtD showed no ALT elevation (Figure 5E). This indicated that the elimination of HBV gtC was most likely mediated by the spontaneous induction of host HBV-specific immune response. Indeed, in four out of five mice infected with AAV-HBV-gtC, low anti-HBs levels were detected (Figure 5F), serum HBV-DNA was very low or negative (Figure 5G), and the mice developed a vigorous CD8 T cell response directed against HBV S (Figure 5H) but not against core or RT(pol) antigens (data not shown).

In three out of four mice infected with AAV-HBV-gtA, low anti-HBs titers (Figure 5F), but no HBV-specific T cells, (Figure 5H) were detected. This explained the selective decrease of HBsAg that is neutralized by the anti-HBs antibodies after infection with AAV-HBV-gtA, while HBeAg levels remained constant because HBV gtA-replicating hepatocytes were not eliminated. As expected, no mice carrying HBV gtB or gtD developed HBV-specific antibodies or T cells (Figures 5F and 5H).

Taken together, our data demonstrate that infecting immunocompetent C57BL/6 mice with AAV-HBV allows for the transduction of hepatocytes with different HBV genotypes and the establishment of persistent HBV replication of HBV gtA, gtB, and gtD but not gtC. HBV carrier mice developed an immune tolerance against HBV gtB and gtD and—at least on a T cell level—also against HBV gtA.

### The *TherVacB* regimen employing MVA-HBVac for boost vaccination induces virus-specific immunity against the most common HBV genotypes

To test the immunogenicity and antiviral efficacy of the *TherVacB* therapeutic vaccine employing MVA-HBVac in mice carrying different HBV genotypes, we vaccinated mice replicating HBV of gtA, gtB, and, as a reference, gtD. Since the establishment of persistent replication with HBV gtC was not successful in immunocompetent C57BL/6 mice, we excluded this genotype from the vaccination studies.

Six weeks after AAV-HBV transduction, *TherVacB* vaccination was initiated (week 0). Mice received two protein vaccinations with adjuvanted HBsAg (gtA) and HBcoreAg (gtD) at weeks 0 and 2, followed by a boost vaccination with MVA-HBVac at week 4 (according to the scheme depicted in Figure 3A). Endpoint analyses were performed 7 weeks later at week 11. Groups of mice infected with AAV-HBV-gtA, -gtB, and -gtD that did not receive vaccinations served as controls.

**Figure 6:**
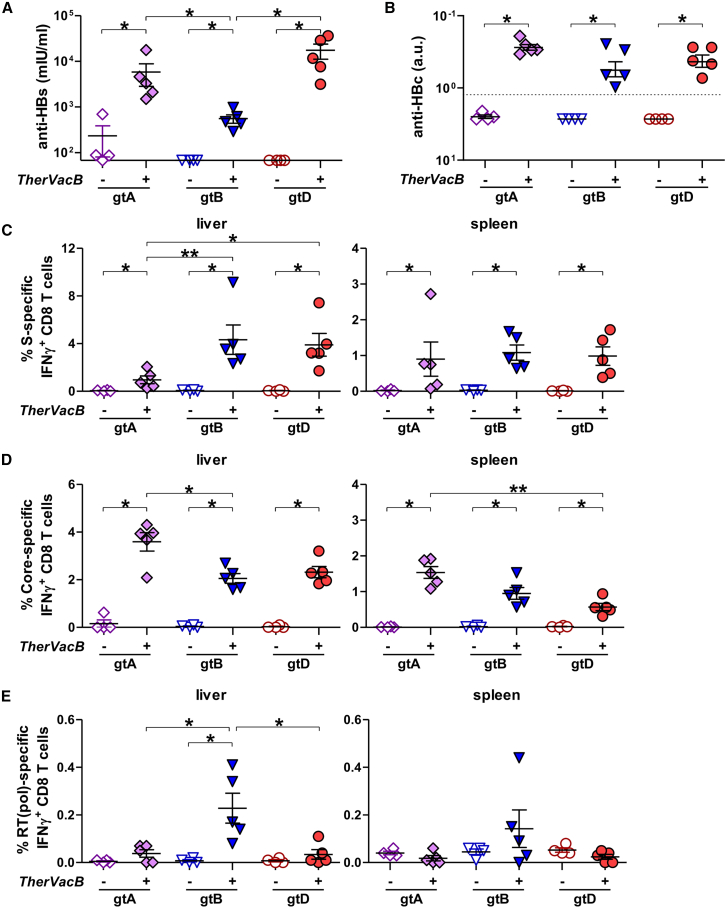
Immunogenicity of *TherVacB* vaccination using MVA-HBVac in mice carrying HBV gtA, gtB, and gtD C57BL/6 mice (*n* ≥ 4) were intravenously injected with AAV vectors carrying a 1.3-fold overlength HBV genome of gtA, gtB or gtD. *TherVacB* vaccination was initiated 6 weeks after AAV-HBV infection (week 0). Mice were vaccinated twice with c-di-AMP-adjuvanted, particulate HBsAg and HBcoreAg (week 0 and week 2) and 2 weeks later boosted with MVA-HBVac (week 4; see Figure 3A). Mice receiving no vaccinations served as controls. Mice were sacrificed, and immune responses were analyzed 7 weeks after the MVA boost (week 11). (A and B) Anti-HBs (A) and anti-HBc (B) levels were determined in mouse serum at the final analysis. (C–E) HBV-specific T cell responses were analyzed in lymphocytes isolated from the spleen and liver by ICS after 16 h *ex vivo* stimulation with overlapping peptide pools. Frequencies of (C) S-specific, (D) core-specific, and (E) RT(pol)-specific IFNγ^+^ CD8 T cells detected in livers and spleens. Mean ± standard error of mean is shown. Statistical analyses were performed using (A–E) Kruskal-Wallis test with Dunn’s multiple comparison correction test. Asterisks mark statistically significant differences: ∗*p* < 0.05; ∗∗*p* < 0.01.

We first compared the induction of HBV-specific antibody and T cell responses at week 11 in all groups of mice. *TherVacB* elicited strong anti-HBs and anti-HBc antibody responses in mice replicating HBV gtA, gtB, and gtD (Figures 6A and 6B). The *TherVacB*-induced anti-HBs titers were 1 log_10_ to 1.5 log_10_ lower in mice replicating HBV gtB than in mice carrying HBV gtA or gtD, but still reached a mean titer of 600 IU/mL. Next, we investigated the effects of the *TherVacB* employing an MVA-HBVac boost on the induction of HBV-specific effector CD8 T cells. As expected, HBV-specific T cell immunity was only observed in vaccinated mice. *TherVacB* vaccination elicited significant S- and core-specific IFNγ^+^ CD8 T cell responses detected in the livers and spleens (Figures 6C and 6D) of mice persistently replicating HBV gtA, gtB, and gtD. However, in mice replicating HBV gtA, more core-specific but fewer S-specific CD8 T cells infiltrated the liver (Figures 6C–6E). Interestingly, *TherVacB* elicited an RT(pol)-specific IFNγ^+^ CD8 T cell response in the HBV gtB carrier mice but only in a few gtA or gtD carrier mice (Figure 6E).

Taken together, the *TherVacB* regimen employing a pan-genotypic MVA-HBVac vaccine vector for boost was capable of breaking virus-specific immune tolerance and inducing potent and broad immunity against HBV of different genotypes.

### Characterization of *TherVacB*-induced hepatic CD8 T cells

To characterize *TherVacB*-induced CD8 T cells isolated from the livers of mice replicating HBV gtA, gtB, or gtD in more detail, we used MHC-K^b^ multimers loaded with the immunodominant peptides covering amino acids 190–198 of S (S_190_) or 93–100 of core (C_93_) that allow for the detection of HBV-specific CD8 T cells independent of their effector functions. We excluded RT(pol)-specific CD8 T cells from analyses due to their low frequencies.

**Figure 7:**
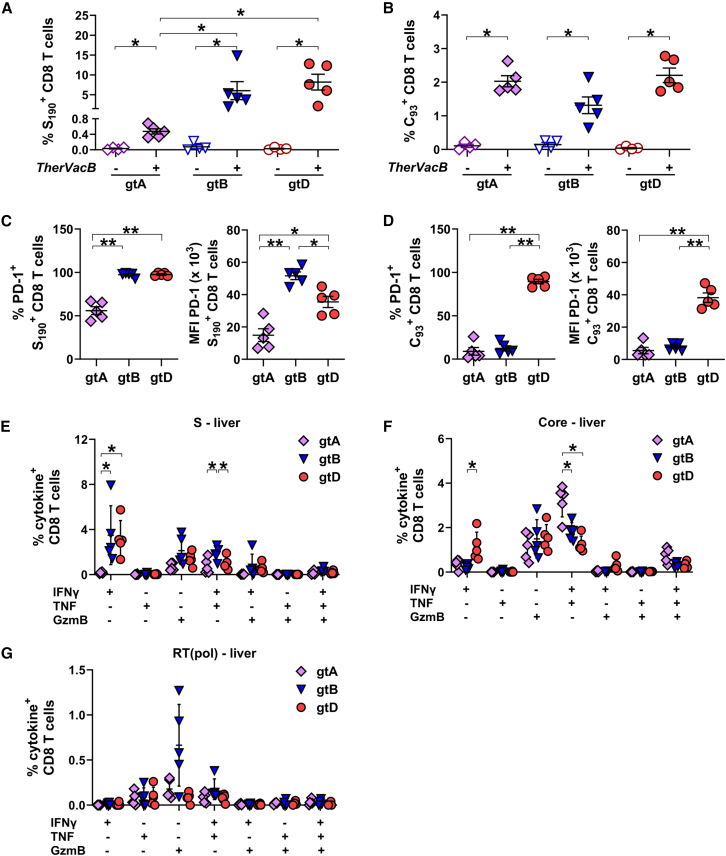
Signature and functionality of intrahepatic HBV-specific CD8 T cells after *TherVacB* vaccination using MVA-HBVac in mice carrying HBV gtA, gtB, and gtD C57BL/6 mice (*n* ≥ 4) were intravenously injected with AAV vectors carrying a 1.3-fold overlength HBV genome of gtA, gtB, or gtD. *TherVacB* vaccination was initiated 6 weeks after AAV-HBV infection (week 0) and mice were 7 weeks after the MVA boost (week 11). (A and B) Frequencies of S-specific (A) and core-specific (B) CD8 T cells directly detected *ex vivo* in hepatic lymphocyte fraction staining with S_190_ or C_93_ multimer, respectively. (C and D) Frequencies and expression levels of PD-1 on liver-associated S-specific (C) and core-specific (D) CD8 T cells. (E–G) Fraction of S-specific (E), core-specific (F), and RT(pol)-specific CD8 T cells isolated from the liver staining positive for indicated cytokines after *ex vivo* stimulation with three optimized overlapping peptide pools. Mean ± standard error of mean is shown. Statistical analyses were performed using (A–D) Kruskal-Wallis test with Dunn’s multiple comparison correction and (E–G) two-way ANOVA. Asterisks mark statistically significant differences: ∗*p* < 0.05; ∗∗*p* < 0.01.

*Ex vivo* staining of liver-infiltrating lymphocytes with S_190_ and C_93_ multimers revealed that all mice receiving *TherVacB* developed significant amounts of S- and core-specific CD8 T cells (Figures 7A and 7B). Only background values were detected in the non-vaccinated animals, confirming that AAV-HBV mice replicating HBV of gtA, B, and D did not develop any HBV-specific CD8 T cell response in the liver without vaccination. While the frequencies of S-specific CD8 T cells were significantly higher in immunized mice replicating HBV gtB and gtD (Figure 7A), the frequencies of C_93_-specific CD8 T cells after *TherVacB* were comparable between the different HBV genotypes (Figure 7B).

Since programmed death receptor-1 (PD-1) plays a key role in the activation and modulation of the effector function of HBV-specific T cells in the liver,^15^ we determined the expression level of PD-1 on *TherVacB*-elicited S- and core-specific CD8 T cells. Although the fraction of PD-1 expressing S_190_^+^ CD8 T cells was comparable in the livers of mice carrying HBV gtB, gtD, but higher than in gtA, the expression level of PD-1 per cell was significantly higher in HBV gtB compared to gtD and lowest in gtA (Figure 7C). The expression level of PD-1 on hepatic-vaccine-induced C_93_^+^ CD8 T cells, however, was only high in mice replicating HBV of gtD (Figure 7D).

We next examined whether *TherVacB*, employing MVA-HBVac, induces polyfunctional-virus-specific hepatic CD8 T cell responses. We detected predominantly IFNγ^+^ mono- and IFNγ^+^TNF^+^ double-positive S- and core-specific CD8 T cells (Figures 7E and 7F). The frequency of functional S-specific hepatic CD8 T cells was significantly higher in HBV gtB carrier mice (Figure 7E), although PD-1 expression levels on S_190_^+^ T cells were the highest for this genotype (Figure 7C). Frequencies of IFNγ^+^TNF^+^ double-positive, C_93_-specific CD8 T cells were significantly increased in mice carrying HBV gtA as compared to gtB and gtD, while the highest fraction of IFNγ^+^ mono-positive CD8 T cells was detected for gtD (Figure 7F). All immunized mice had a fraction of S- and core-specific CD8 T cells staining positive for the cytotoxic molecule granzyme B (GzmB) (Figures 7E and 7F) compared to only background values detected in the corresponding unvaccinated controls (data not shown). Interestingly, four out of five mice carrying HBV gtB were positive for RT(pol)-specific GzmB^+^ CD8 T cells after the boost with MVA-HBVac (Figure 7G), confirming the induction of a low RT(pol)-specific CD8 T cell response in gtB carriers.

Taken together, these data indicate that HBV-specific CD8 T cells induced by *TherVacB* employing MVA-HBVac retain their effector function upon contact with viral antigens in the liver, but the magnitude and specificity of the CD8 T cell response are shaped by the replicating HBV genotype.

### *TherVacB*, employing MVA-HBVac as a booster, effectively controls HBV of the most common genotypes

**Figure 8:**
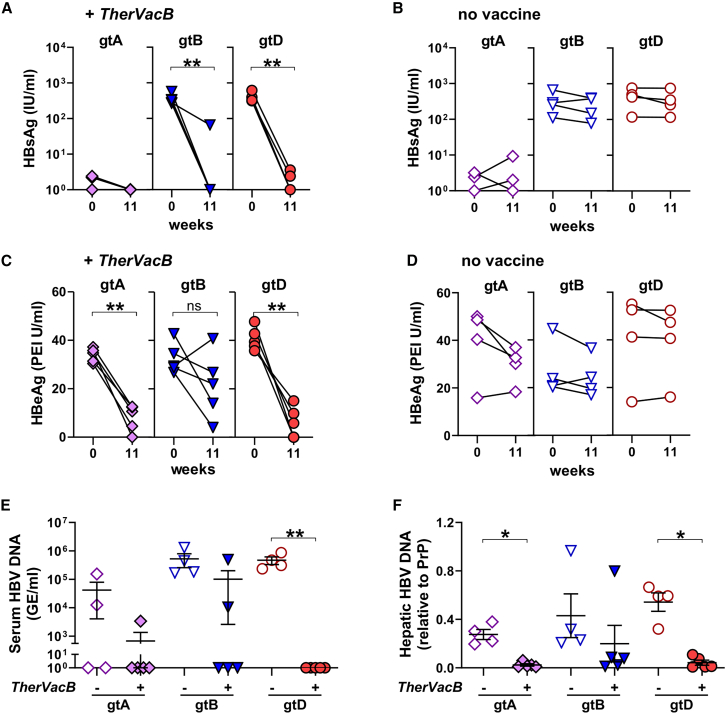
Antiviral effect of *TherVacB* vaccination using MVA-HBVac in mice carrying HBV gtA, gtB, and gtD C57BL/6 mice (*n* ≥ 4) were intravenously injected with AAV vectors carrying a 1.3-fold overlength HBV genome of gtA, gtB, or gtD. *TherVacB* vaccination was initiated 6 weeks after AAV-HBV infection (week 0), and mice were 7 weeks after the MVA boost (week 11). (A and B) Serum HBsAg levels in the serum before vaccination and at the final analysis in mice receiving *TherVacB* (A) and in unvaccinated controls (B). (C and D) Serum HBeAg levels before vaccination and at the final analysis in mice receiving *TherVacB* (C) and in unvaccinated controls (D). (E and F) Quantification of serum HBV DNA (E) and intrahepatic HBV DNA (F) in the liver tissue at final analysis. Mean ± standard error of mean is shown. Statistical analyses were performed using (B and D) Mann-Whitney test and (E and F) Kruskal-Wallis test with Dunn’s multiple comparison correction. Asterisks mark statistically significant differences: ∗*p* < 0.05; ∗∗*p* < 0.01. GE, genome equivalents.

Next, we determined whether *TherVacB* with MVA-HBVac boost can break immune tolerance and control the persistent replication of HBV of the most prevalent genotypes. A *TherVacB*-mediated decrease in serum HBsAg to low or undetectable levels was observed in all mice at week 11, except for one outlier mouse from the HBV gtB group, in which HBsAg only dropped by 1 log_10_ (Figure 8A). As expected, no significant changes in serum HBsAg levels were observed without vaccination (Figure 8B). Importantly, serum HBeAg, indicating persistent replication of HBV in the liver, also significantly dropped in all vaccinated mice irrespective of whether they replicated HBV gtA, gtB, or gtD (Figure 8C) while it remained high in unvaccinated controls (Figure 8D). In 12 out of 15 immunized mice, serum HBV DNA levels dropped below the limit of detection, and another two mice experienced a 2log_10_ decrease compared to unvaccinated controls (Figure 8E). Accordingly, a significant reduction in intrahepatic HBV DNA levels was observed in 14 out of 15 immunized mice (Figure 8F). In one outlier mouse from the gtB group, HBeAg unexpectedly increased despite vaccination, no decline in intrahepatic HBV DNA, neither in serum nor in the liver, and only a low T cell response was detected (Figures 8C–8F), indicating that vaccination did not work properly.

Taken together, including MVA-HBVac that covers the major antigens of different HBV genotypes into the *TherVacB* regimen effectively suppressed the replication of the most common genotypes of HBV in mice.

## Discussion

An effective and broadly applicable therapeutic hepatitis B vaccine requires overcoming strong HBV-specific B and T cell tolerance against HBV of different genotypes and serotypes. Here, we report the successful generation, characterization, and *in vivo* validation of a novel therapeutic vaccine vector against chronic hepatitis B, MVA-HBVac, designed to target >95% of the circulating HBV isolates worldwide.^18^ To demonstrate the broad efficacy of MVA-HBVac, we established immunocompetent HBV carrier mouse models for HBV gtA, gtB, and gtD. We demonstrate that the application of MVA-HBVac as a boost vector is not only safe and immunogenic in mice persistently replicating HBV gtD, which is the most prevalent genotype in Europe, Russia, and the Far East, but also elicits an antiviral effect within the therapeutic vaccination scheme *TherVacB* to the other relevant HBV genotypes, namely gtA, which is highly prevalent in North America and Europe and East Africa, and gtB, which shares most immunodominant epitopes in humans with HBV gtC and is most prevalent in South-East Asia.^18^ In addition, we demonstrated that HBV-naïve mice develop a more prominent T cell response after *TherVacB* than the HBV carrier mice, confirming that immune tolerance develops in HBV carrier mice.

*TherVacB* was designed to activate potent effector HBV-specific B, CD4, and CD8 T cell responses to target all branches of the dysfunctional adaptive immune response observed in chronic HBV infection in humans. Boosting with recombinant MVA specifically aims to expand virus-specific CD8 T cell responses,^17^^,^^26^ essential to eliminating infected hepatocytes and mediating the clearance of CHB in patients.^27^ MVA, a vector of choice to boost T cell responses, is characterized by an excellent safety profile, which facilitates the use of the vaccine not only in chronically infected but also in immunosuppressed individuals, such as HIV/HBV co-infected patients.^28^ To broaden the spectrum of *TherVacB*-mediated cellular immunity, we designed MVA-HBVac based on the sequences of the four major HBV proteins, the S and L envelope, core, and polymerase proteins, taking into account that strong immunity against these three antigens is crucial for the resolution of acute hepatitis B.^3^ Although the use of therapeutic vaccines based on HBV X protein has also been proposed,^29^ X protein was not included in our construct due to safety concerns related to its anti-apoptotic and potentially oncogenic properties.^30^^,^^31^

To cover different HBV serotypes and all major epitopes of genotypes A to E, accounting for >95% of all HBV infections,^18^ presented on the highly prevalent HLA types of humans worldwide, we selected sequences of five strongly matching HBV proteins to generate the optimized HBVac insert. The sequences selected are identical to naturally occurring viral sequences to ensure correct folding. This ensures efficient antigen processing and presentation on infected cells and secretion of the highly immunogenic non-infectious subviral particles.^19^ To allow for a combination of the different protein-coding genes in one MVA vector, we designed a polycistronic insert taking advantage of phage-derived 2A peptides that serve as ribosomal switch sites. The 2A peptides used to combine the HBV proteins were selected according to their reported *in vivo* efficacy and should result in the expression at equimolar ratios of all five proteins encoded by the polycistronic insert.^20^ Although we demonstrated that all single proteins were expressed as expected, we cannot exclude the possibility of an unsuccessful ribosome skipping at one of the 2A sites and the expression of fusion proteins consisting of two linked viral proteins *in vivo*. This should, however, not have negative effects on vaccine efficacy and safety. HBV subviral particles, formed by the S protein, and VLPs based on the HBV core protein were successfully employed as vaccine carriers carrying various antigens,^32^^,^^33^ and the potential expression of fusion proteins by MVA-HBVac may even improve the overall vaccine immunogenicity.

We previously demonstrated that the *TherVacB* vaccination scheme consists of two prime vaccinations using a combination of VLP HBsAg derived from yeast and HBcoreAg derived from *Escherichia coli*, and a suitable adjuvant,^17^ followed by a boost vaccination with a combination of two recombinant MVAs, expressing either HBV S or core protein, is capable of breaking virus-specific immune tolerance and achieving long-term antiviral control of HBV in preclinical mouse models.^14^^,^^16^^,^^17^ By selectively depleting CD4 T cells during the protein immunizations, we previously demonstrated that *TherVacB* priming is primarily responsible for eliciting HBV-specific B cell and CD4 responses.^17^ Since the priming remained the same, employing a boost with the polycistronic MVA-HBVac vector did not significantly influence anti-HBs and anti-HBc titers or the magnitude of S- and core-specific IFNγ CD4 T cell responses. However, using MVA-HBVac for boost vaccination allowed the induction of RT(pol)-specific IFNγ CD4 T cell responses in HBV-naïve mice. As no RT protein was used for priming, these results demonstrated the excellent *in vivo* immunogenicity of the novel MVA-HBVac vector. Consistently, robust CD8 T cell immunity against S and core antigens, but also a weak RT-specific CD8 T cell response, was detected in HBV-naïve mice.

In HBV-carrier mice, MVA-HBVac boosting also induced a vigorous, comparable S- and core-specific CD8 T cell response, although the magnitude of response was lower compared with HBV naive mice. Moreover, in most of the experiments performed in HBV carrier mice, no RT(pol)-specific CD4 and CD8 T cells could be detected, which overall is weaker due to the lack of priming immunization against RT(pol). This demonstrates the establishment of HBV-specific immune tolerance after AAV-HBV infection, even in fully immune-competent mice, where hepatocytes continuously express HBV proteins, negatively impacting T cell activation and functionality.^34^ This is most likely due to the fact that AAV-8 used to generate AAV-HBV specifically targets hepatocytes but not dendritic or Kupffer cells, and thus HBV-specific T cells are not efficiently primed.^35^^,^^36^ In addition, T cells recognizing their antigen on hepatocytes establish close and extensive contact with liver sinusoidal endothelial cells, thereby enhancing adenylyl cyclase-cAMP-PKA signaling in the T cells that remain in the liver because they recognize their cognate antigen locally in the liver. This results in a block of T cell receptor activation by this liver immune rheostat.^37^

The failure to induce RT(pol)-specific T cells in the majority of HBV-carrier mice further confirms our previous reports,^17^ which underline the importance of the appropriate priming and boosting of immune responses by *TherVacB* for the immune control of persistent HBV infection. Thus, employing a vaccine platform, e.g., DNA- or RNA-based vaccines, that allows for priming against multiple antigens, including RT(pol) and matching those encoded by MVA-HBVac, might be an interesting alternative to further broaden the *TherVacB*-induced T cell responses. Interestingly, mice carrying HBV of genotype B were able to mount a weak RT(pol)-specific IFNγ- and GzmB-positive CD8 T cell response in the liver and spleen, in contrast to the mice replicating genotypes A or D. This suggests that the effector function of CD8 T cells upon therapeutic vaccination might rely on the extent of antigen presentation, which may depend on the sequence variability between the distinct HBV strains.

Although the AAV-HBV infection allows for the establishment of an HBV carrier model that shows a distinct level of immune tolerance and has proven invaluable for the development of potentially curative treatment approaches, it can only partially predict the treatment outcome in patients: HBV cannot spread in mice, the duration of infection in mice and chronically infected patients is very different, and the immune response in inbred mice is more restricted as our laboratory mouse strains have a different and very narrow MHC repertoire.^38^ For example, in contrast to our HBV-carrier mice, patients with chronic HBV infection show a certain level of endogenous HBV-Pol-specific T cells,^39^ which may be boosted by the MVA-HBVac vaccination. Unlike our HBV-carrier mice, chronically infected patients develop anti-HBc antibodies. Thus, unraveling the full potential of MVA-HBVac boost in inducing genotype- and serotype-specific CD8 T cell responses will only be possible in patients.

Unfortunately, we could not assess the efficacy of the vaccination in AAV-HBV mice replicating HBV of gtC. Surprisingly, infection of C57BL/6 mice with AAV-HBV gtC resulted in self-limiting infection, accompanied by spontaneous induction of anti-HBs and S-specific CD8 T cell response detectable in liver and spleen. This virus-specific immunity was capable of eliminating HBV-positive hepatocytes, as marked by loss of circulating HBV antigens and DNA, and was accompanied by an ALT flare. The spontaneous formation of HBV-specific cytotoxic T cell responses we detected after infection transduction with HBV gtC, in contrast to the other HBV genotypes, suggests that the immune response self-limits the infection. This is in contrast to HBV-gtC infection in humans, which may be persistent. Previous studies have reported the successful establishment of persistent replication of HBV gtC in immunocompetent C57BL/6 mice using hydrodynamic injection of HBV-encoding plasmids.^40^ Age, sex, and genetic background of the mouse strain, the HBV sequence, and the applied dose potentially influencing persistence^41^ were similar to our experiments. We can thus only speculate that the AAV-HBV-gtC-induced T cell response did not allow for the development of persistent HBV replication, while the other examined HBV constructs did.

Longitudinal and sustained reduction of HBV parameters in serum is crucial for successful therapeutic vaccination against CHB, as indicated by the seroconversion of HBsAg to anti-HBs, which defines a functional cure in chronic HBV carriers.^42^ Here, we demonstrate that the *TherVacB* regimen employing MVA-HBVac effectively reduced serum HBsAg and HBeAg levels in mice persistently replicating HBV, not only of gtD but also of gtA and gtB. Consistent with our previous reports,^17^ reduction in serum HBsAg is mainly mediated by high levels of anti-HBs induced during priming with recombinant, particulate HBsAg. Maintaining HBsAg levels below the detection limit over time, however, was supported by a strong HBV-specific CD8 T cell response and the elimination of HBsAg-positive HBV-infected cells.^43^ The reduction in serum HBeAg, which in the AAV-HBV mouse model is the best marker of HBV replication in the liver, occurred shortly after the MVA boost and was accompanied by a mild ALT flare, indicating that MVA-HBVac effectively elicited a cytotoxic CD8 T cell response. The ALT increase was mild and only transient, indicating that vaccination with MVA-HBVac is well tolerated but also suggesting that the strong antiviral effect observed in this study may at least be partially attributed to a cytokine-mediated elimination of HBV in a non-cytolytic fashion.^44^^,^^45^

Taken together, our results demonstrate that the novel pan-genotypic MVA-HBVac vector is highly immunogenic and efficacious in mice persistently replicating HBV when used to boost the T cell response after protein-prime applying the *TherVacB* regimen. MVA-HBVac not only broadened the efficacy of *TherVacB* to most of the relevant circulating HBV genotypes worldwide but also is a single vector, facilitating clinical translation. This allowed us to initiate a phase 1 clinical trial in 2024 using MVA-HBVac and the *TherVacB* vaccination scheme.

## Materials and methods

### Cell culture

Chicken embryo fibroblasts (CEFs) were isolated from 11-day-old, embryonated chicken eggs and cultured in VP-SFM media (GIBCO) with 1% Penicillin/Streptomycin (Sigma-Aldrich), 1% non-essential amino acids (Sigma-Aldrich), and 1% L-Glutamine (Sigma-Aldrich) at 37°C, 5%CO2. DF-1 cells were cultured using DMEM+GlutaMAX (GIBCO) supplemented with 10% FCS (Thermo Fisher Scientific Inc), 1% Penicillin/Streptomycin (GIBCO), 1% sodium pyruvate (GIBCO), 1% non-essential amino acids (GIBCO), and 200mmol/L L-Glutamine (GIBCO) at 37°C, 5% CO2. HeLa cells were cultured in DMEM (GIBCO) supplemented with 10% FCS, 1% Penicillin/Streptomycin, 1% sodium pyruvate, 1% NEAA, and 200 mmol/L L-Glutamine at 37°C, 5% CO2.

### Generation of MVA-HBVac

MVA-HBVac was generated according to an established protocol.^21^ Briefly, the HBVac insert was cloned into a pIIIH5redK1L vector plasmid using the BamHI and PmeI restriction sites. The vector was transfected into MVA-F6-infected chicken embryo fibroblasts (CEFs), leading to the insertion of the HBVac insert in deletion site III of the viral genome via homologous recombination and creation of the recombinant MVA-HBVac. Red plaques indicated successful integration of the HBVac fragment into the genome of MVA. MVAred-HBVac was further passaged to get rid of the marker protein. The purity and identity of MVA-HBVac was verified by using 6-deletion PCR as previously described.^21^

### Virus amplification

MVA F6 and MVA-HBVac were concentrated and purified according to a modified protocol.^21^ Briefly, BHK-21 cells were infected using MVA-HBVac for 48 h. Then, infected cells were scraped from flasks, subjected to three freeze-thaw cycles, sonicated three times for 30 s, and then centrifuged to remove cell debris. The virus contained in the supernatant was further purified and concentrated by two consecutive ultracentrifugation steps through a 36% sucrose cushion at 13,500 rpm, 4°C for 1.5 h (Optima L 90K, Beckman Coulter GmbH; rotors: SW32TI, SW 41TI). After centrifugation, the remaining virus pellet was dissolved in 1 mM Tris HCL, pH9, and stored at −80°C until use.

### Determination of viral growth

DF-1 and HeLa cells were seeded into 12-well plates and incubated for 24 h. After reaching 80% confluency, cells were infected with MVA-HBVac or MVA F6 at MOI 0.1. Cells were harvested at the indicated time points, and viral load was determined using a plaque assay as described in the article by Kremer et al.^21^ Briefly, DF-1 cells were seeded into 6-well plates at a density of 2 × 10e6 cells per well, incubated overnight, and infected using a series of 10-fold dilutions of the virus stock preparation. After 2 h, cells were washed with PBS and further incubated with media for 48 h. Then cells were fixed for 5 min with an ice-cold mix of acetone/methanol in a ratio 1:1, washed with PBS, and incubated in blocking buffer (PBS with 3% FCS) for 1 h at room temperature or at 4°C overnight. Viral plaques were detected using rabbit anti-Vaccinia virus (Lister strain) antibody (Acris Antibodies GmbH) and POD-conjugated anti-rabbit IgG (H + L) antibody (Jackson Immuno Research), which were diluted in blocking buffer (1:2,000 and1:5,000, respectively). After the final wash, viral plaques were visualized by using KPl TrueBlueTM solution (Sera Care; cat. no. 5510.0030) and counted.

### Ethical statement

All animal experiments were performed according to the guidelines from the European Health Law of the Federation of Laboratory Animal Science Associations (FELASA), the German regulations of the Society for Laboratory Animal Science (GV-SOLAS) as well as the 3R rules and approved by the local Animal Care and Use Committee of Upper Bavaria (permission number: ROB-55.2-1-54-2532-112-13 and 55.2-2532.Vet_02-18-24). Animals were kept in a specific pathogen-free animal facility, and all experiments followed institutional guidelines. All efforts were made to ensure the well-being of the animals and minimize any suffering.

### Animal models and AAV transduction

Eight to ten weeks old male C57BL/6, haplotype H-2b/b wild-type mice were purchased from Janvier Labs (Le Genest-Saint-Isle, France). Infection with AAV-HBV was performed intravenously 6 weeks before the start of the therapeutic vaccination to enable persistent HBV replication. Mice were infected with either 4 × 10e9 genome equivalent (GE) AAV-HBV1.2 containing a 1.2-fold overlength HBV genome of gtD^23^ or AAV-HBV1.3 encoding for 1.3-fold overlength HBV genome of gtA, gtB, gtC, or gtD.^25^ The doses of all AAV-HBV1.3 were adjusted to obtain comparable infection levels between the different genotypes. HBeAg and HBsAg titers of all mice were determined 2 days before vaccination, and the animals were allocated according to their titers to create comparable groups.

### *TherVacB* vaccination

Animals were vaccinated using a modified *TherVacB* protocol of the previously reported vaccination scheme.^14^ Naive C57BL/6 mice received one intramuscular (i.m.) protein vaccination consisting of 20 μg each of recombinant, particulate HBsAg (gtA, adw), and HBcoreAg (gtD, ayw, kindly provided by APP Latvijas Biomedicinas, Riga, Latvia) at week 0. AAV-HBV mice received two protein vaccinations with 10 μg each of HBsAg and HBcoreAg at weeks 0 and 2. Vaccine antigens were adjuvanted with 10 μg of cyclic di-adenylate monophosphate (c-di-AMP) or 30 μg of CpG-1018 (InvivoGen, San Diego, CA). Two weeks after protein vaccination, the mice received boosting vaccination with either a mixture of 3 × 10e7 infectious units (IFUs) each of recombinant MVA expressing HBV S or HBV core protein (both gtD, ayw) or polycistronic MVA-HBVac.

### Flow cytometry analysis

Liver-associated lymphocytes (LALs) and splenocytes were isolated and stained using MHC class I multimers or by ICS as previously described.^16^^,^^17^^,^^43^ HBV-specific CD8 T cells were detected through staining with PE-labeled MHC class I (H-2K^b^) multimers conjugated with the H-2K^b^-restricted, S-derived peptide S_190–197_ (S_190_; VWLSAIWM) or core-derived peptide C_93–100_ (C_93_, MGLKFRQL) or S190. To determine the background values, staining with a multimer conjugated with the ovalbumin-derived peptide S8L_257-264_ (OVA_S8L_, SIINFEKL) was performed.

For ICS, isolated T cells were *ex vivo* stimulated overnight with 1 μg/mL of synthetic overlapping peptide pools covering either the single antigens of the HBVac insert (five peptide pools), three optimized peptide pools comprising the three immunogens S, core and RT(pol) while avoiding duplicate activation of T cells by the overlapping antigen sequences of the different HBV genotypes, or OVA_S8L_ peptide as a control. Stimulation was performed in the presence of 1 mg/mL brefeldin A (Sigma-Aldrich).

Cells were stained extracellularly using anti-CD4-APC (clone GK1.5, eBioscience), anti-CD8-PacificBlue (clone 53-6.7, BD Pharmingen), and anti-PD-1-PerCP-eF710 (clone J43, eBioscience) antibodies. Dead cells were excluded from analyses using Fixable Viability Dye (eFluor780; eBioscience). Afterward, the cells were fixed and permeabilized with Cytofix/Cytoperm Kit (BD Bioscience) and stained intracellularly using anti-IFNγ-FITC (clone XMG1.2; BD Pharmingen), anti-TNF-PE-Cy7 (clone MP6-XT22, BD Pharmingen), anti-IL-2-APC (clone JES6-5H4, eBioscience), or the cross-reactive anti-human GzmB-APC (clone GRB04; Invitrogen, Carlsbad, CA) antibodies. Data were acquired using a CytoFlexS flow cytometer (Beckmann Coulter) and analyzed using FlowJo software version 10 (Tree Star).

Data from multimer and intracellular cytokine staining are presented as relative values after subtracting background obtained using the OVA_S8L_ peptide. Nonspecific T cell frequencies detected for LALs staining with multimers were, on average, 0.15% ± 0.08%. For ICS, the background values were as follows: IFNγ^+^ CD4 T cells in the spleen, 0.01% ± 0.01%; IFNγ^+^ CD4 T cells in the liver, 0.04% ± 0.03%; CD8 T cells in the spleen—IFNγ^+^, 0.10% ± 0.06%; TNF^+^, 0.33% ± 0.15%; IL-2^+^, 0.04% ± 0.03%; CD8 T cells in the liver—IFNγ^+^, 0.22% ± 0.09%; TNF^+^, 0.25% ± 0.07%; GzmB^+^, 0.68% ± 0.20%.

### Serological and virological analysis

Serum titers of viral antigens HBsAg and HBeAg were determined on the Architect platform (Abbott Laboratories) using quantitative reagent kits (Ref.:6C36-44; Ref.: 6C32-27) and HBeAg quantitative calibrators (Ref.: 7P24-01). Anti-HBs antibodies were quantified using the Architect platform and anti-HBs tests (Ref.: 7C18-27). Anti-HBc was measured using Roche Cobas e402 (Roche Diagnostics, Basel-Kaiseraugst, Switzerland) or Liaison (DiaSorin, Saluggia, Italy) platforms. ALT levels were determined in serum diluted 1:4 with PBS using a Reflotron GPT/ALT test (Roche, Mannheim, Germany).

DNA was extracted from 25 mL serum using the QIAamp MinElute Virus Spin Kit (Qiagen, Hilden, Germany) according to the manufacturer’s protocol and eluted into 35 μL H_2_O and from 20 mg of liver tissue using the NucleoSpin Tissue DNA Kit (Macherey-Nagel, Dueren, Germany) according to the manufacturer’s instructions. The quantification of HBV-DNA was performed through real-time PCR with SYBR-Green, as previously described.^24^ Quantitative values were corrected by subtracting background values obtained in HBV-naïve C57BL/6 mice.

### Immunohistochemistry

Liver tissue samples were fixed in 4% buffered formalin for 48 h and embedded in paraffin. Immunohistochemistry was performed with an anti-HBcore antibody (Diagnostic Biosystems, Pleasanton, CA; 1:50 dilution) according to a protocol described previously.^24^ HBcore-positive hepatocytes were determined in 10 random view fields (20× and 40× magnification) and quantified per mm^2^.

### Statistical analysis

Statistical analyses were performed using GraphPad Prism version 5 (GraphPad Software Inc., San Diego, CA). Statistical differences were analyzed using the Kruskal-Wallis test with Dunn’s multiple comparison correction, two-way ANOVA with Bonferroni multiple comparison correction, or the Mann-Whitney test. *p* values <0.05 were considered significant.

## Data availability

The data generated and/or analyzed during the present study are available from the corresponding author upon reasonable request.

## Acknowledgments

We acknowledge the support of the late Gerd Sutter (†2023), who provided essential reagents and technical know-how for MVA production. The authors thank Susanne Miko, Theresa Asen, and Philipp Hagen for their excellent technical assistance. The research in this study was funded by the 10.13039/501100001659German Research Foundation via TRR179 (project No. 272983813 to U.P. and A.K.), the PoC initiative by the Helmholtz and Fraunhofer Associations (to U.P.), the EU Horizon 2020 consortium *TherVacB**,* the 10.13039/100009139German Center for Infection Research via project TTU 05.803 “HBV Cure”, and the German Ministry for Education and Research (BMBF) via the *TherVacB* PLUS project and C-NATM - Cluster for Nucleic Acid Therapeutics Munich funded in the Clusters4Future initiative, Funding No. 03ZU1201BA. H.A.K. received an MD scholarship from German Center for Infection Research (10.13039/100009139DZIF).

## Author contributions

A.D.K. and U.P. designed the study; A.D.K., M.K., H.A.K., M.M.H., E.A.O., M.G., K.S., and J.S. performed the experiments; A.D.K., M.K., and C.M. analyzed the data; C.K. and J.F. generated recombinant AAVs; C.M., K.S., A.V., M.L., and P.K. provided essential reagents and assistance; U.P., P.K., and A.V. provided supervision and funding; A.D.K., M.K., P.K., and U.P. wrote and finalized the manuscript. All authors read and approved the final version of the manuscript.

## Declaration of interests

U.P., A.D.K., M.M.H., P.K., and T.B. are named as inventors on a patent application describing the therapeutic vaccination scheme of *TherVacB* (PCT/EP2017/050553) and the vector MVA-HBVac. U.P. is a co-founder, shareholder, and board member of SCG Cell Therapy and serves as an ad hoc advisor to Aligos, Arbutus, GSK, Roche, Sanofi, Vaccitech/Barinthos, and Vir Bio. P.K. serves as a scientific advisor for Therawis Diagnostics. The remaining authors declare no competing interests. The funders had no role in the study’s design; in the collection, analyses, or interpretation of data; in the writing of the manuscript; or in the decision to publish the results.

## Supplemental information

Document S1. Figures S1 and S2

Document S2. Article plus supplemental information
