# Supplementary material for: MVA-HBVac—A novel vaccine vector that allows pan-genotypic targeting of hepatitis B virus by therapeutic vaccination
Source: Mol Ther Nucleic Acids. 2025 Jul 23;36(3):102641. doi: 10.1016/j.omtn.2025.102641 (PMC12341611; doi:10.1016/j.omtn.2025.102641)
Supplement: Document S1. Figures S1 and S2 [file mmc1.pdf]

OMTN, Volume *36*

Supplemental information

MVA-HBVac—A novel vaccine vector that allows pan-genotypic targeting of hepatitis B virus by therapeutic vaccination

Anna D. Kosinska, Martin Kächele, Helene A. Kerth, Martin Mück-Häusl, Edanur Ates Öz, Merve Gültan, Lea Hansen-Palmus, Julia Sacherl, Chunkyu Ko, Julia Festag, Michael H. Lehmann, Carolin Mogler, Katja Steiger, Percy A. Knolle, Tanja Bauer, Asisa K. Volz, and Ulrike Protzer

Figure S1

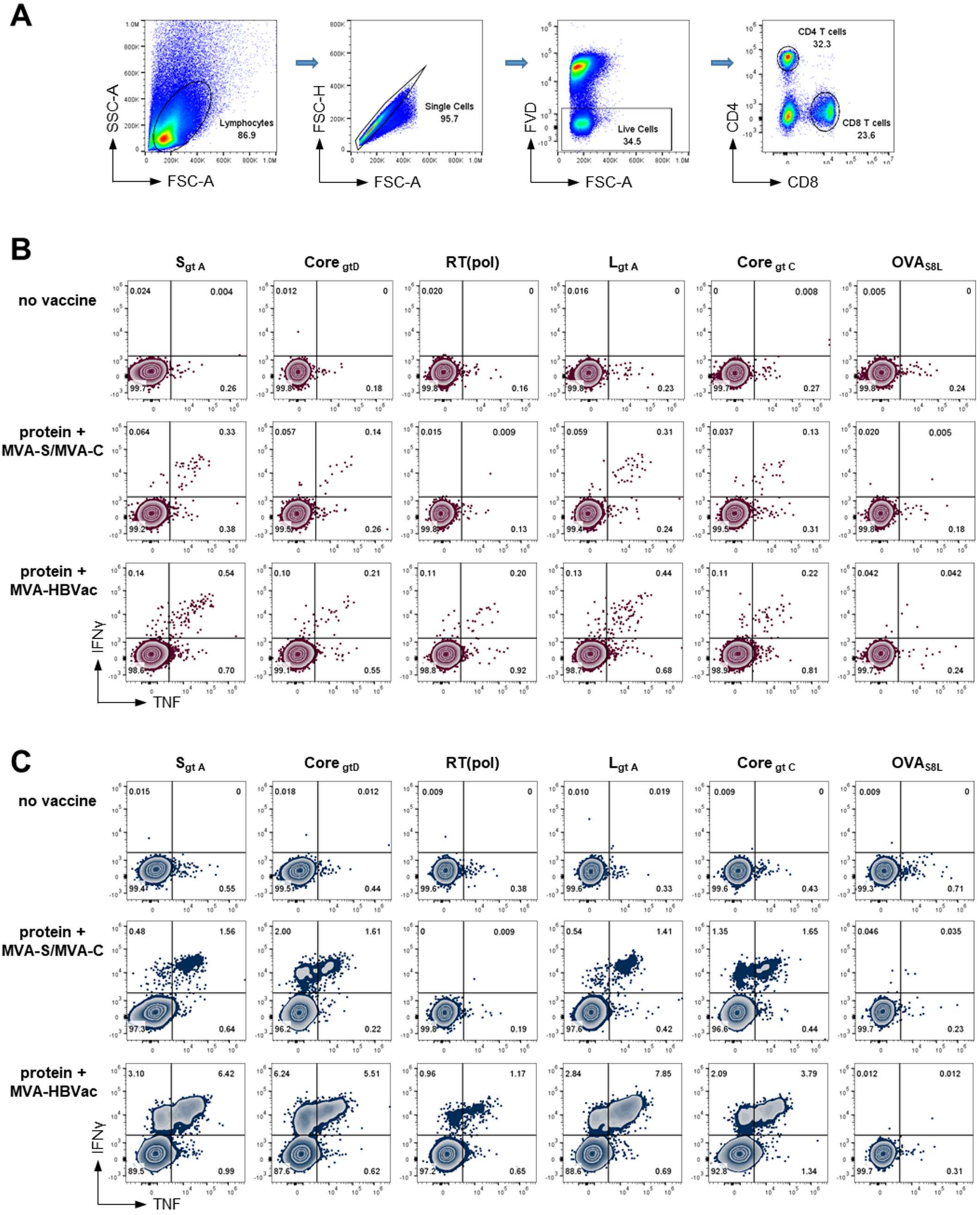

Figure S1. Gating strategy and representative results of flow cytometric analysis. (A) The lymphocyte population was defined using forward-scattered-light-area (FSC-A) and side-scattered-light-area (SSC-A) parameters. Doublets were excluded from the lymphocyte population by plotting forward-scattered-light height (FSC-H) against area (FSC-A) and gating on the diagonal

population. Dead cells were excluded from analysis by gating on fixable viability dye (FVD-eFluor780-A)-negative cells. CD4 or CD8 T cells were gated within the viable lymphocyte population using CD4 APC-A and CD8 PB450-A. (B-C) Representative dot-plots of HBV-specific IFNγ$^+$ TNF$^+$ CD4 (B) and IFNγ$^+$ TNF$^+$ CD8 T cells (C) detected by intracellular cytokine staining (ICS) of isolated splenocytes after 16h *ex vivo* stimulation with 5 overlapping peptide pools covering the proteins expressed by MVA-HBVac. Positioning of gates was determined using controls stimulated with ovalbumin (OVA)-derived peptide S8L$_{257\text{-}264}$ (OVA$_{S8L}$; SIINFEKL). A–area, H – height.

Figure S2

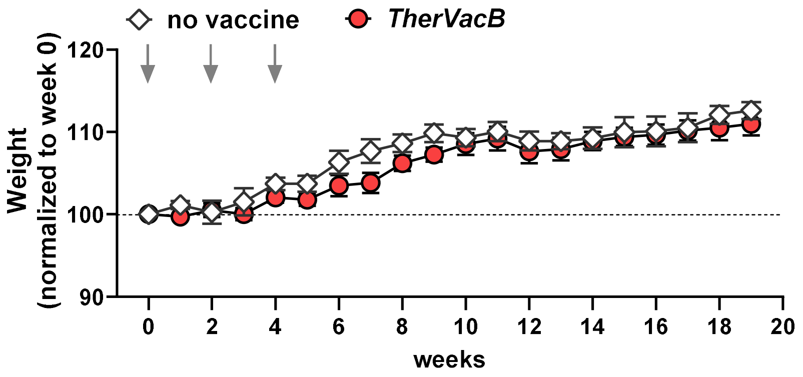

Figure S2. Monitoring of mouse weight. C57BL/6 mice (n=5) were intravenously injected with 4x10e9 GE of an AAV vector carrying a 1.2-fold overlength HBV genome of gtD. *TherVacB* vaccination was initiated 6 weeks after AAV-HBV infection (week 0), and mice were sacrificed 15 weeks after the MVA-HBVac boost (week 19). The body weight of the mice was monitored weekly. The presented values from the beginning of vaccination (week 0) until the end-analysis (week 19) were normalized according to the baseline values at week 0. Arrows indicate the time points of vaccination.
